# Dual Antibiotic and Diffusible Signal Factor Combination Nanoliposomes for Combating *Staphylococcus epidermidis* Biofilm

**DOI:** 10.34172/apb.2021.077

**Published:** 2020-10-14

**Authors:** Golara Gerayelou, Bahman Khameneh, Bizhan Malaekeh-Nikouei, Asma Mahmoudi, Bibi Sedigheh Fazly Bazzaz

**Affiliations:** ^1^School of Pharmacy, Mashhad University of Medical Sciences, Mashhad, Iran.; ^2^Department of Pharmaceutical Control, School of Pharmacy, Mashhad University of Medical Sciences, Mashhad, Iran.; ^3^Nanotechnology Research Center, Pharmaceutical Technology Institute, Mashhad University of Medical Sciences, Mashhad, Iran.; ^4^Biotechnology Research Center, Pharmaceutical Technology Institute, Mashhad University of Medical Sciences, Mashhad, Iran.

**Keywords:** Liposome, Biofilm, Biofilm dispersion, *Staphylococcus epidermidis*, Vancomycin, Cis 2-decenoic acid

## Abstract

***Purpose:*** Microbial biofilms are one of the main causes of persistent human infections. Encapsulation of an antibiotic and a biofilm dispersal agent within a nano-carrier has been recognized as a novel approach to combat the problem of biofilm-related infections. Here, we develop the nanoliposomal formulation for delivery of vancomycin in combination with cis-2- decenoic acid (C2DA), to *Staphylococcus epidermidis* biofilm. The effects of the formulations were studied at two stages: biofilm growth inhabitation and biofilm eradication.

***Methods:*** Liposomal formulations were prepared by the solvent evaporation dehydration-rehydration method and were evaluated for size, zeta potential, and encapsulation efficacy. The ability of different agents in free and encapsulated forms were assessed to evaluate the anti-biofilm activities.

***Results:*** Vancomycin and C2DA were successfully co-encapsulated in the same nanoliposome (liposomal combination). The zeta potential values of the liposomal formulations of vancomycin, C2DA, and the liposomal combination were 37.2, 40.2, 51.5 mV, and the mean sizes of these liposomal formulations were 167.8±1.5, 215.5±8.8, 235.5±0.01, respectively. Encapsulation efficacy of C2DA was 65% and about 40% for vancomycin. The results indicated that liposomal combination exerted strong anti-biofilm activities, slightly exceeding those observed by the free form of a combination of vancomycin and C2DA, but higher than either agent used alone in their free forms. The anti-biofilm activity of formulations followed concentration and time-dependent manner.

***Conclusion:*** The combination of vancomycin and C2DA could inhibit biofilm formation. Employing the liposomal combination is a considerable method to remove bacterial biofilm.

## Introduction

Prosthetic devices have been extensively used in clinical applications. The usage of these devices is restricted in some cases due to the high risk of bacterial infections accompanied by them.^1^ These types of infections frequently do not respond to antimicrobial agents, therefore, removing the device is often required. The genus of Staphylococcus including *Staphylococcus epidermidis* and *Staphylococcus aureus* are the most common microorganisms causing device-related infections.^2,3^ One of the major reasons for this antibiotic treatment failure is the formation of bacterial biofilm on the surfaces of indwelling medical devices within which bacteria are protected from the attack of antibacterial agents and host-defensive mechanisms.^4,5^

To date, various approaches have been reported for treating bacterial biofilm infections involving combination therapy, application of dispersing agents or employing nanoparticulate systems.^6-10^

It has been reported that cis-2-decenoic acid (C2DA), a medium-chain fatty acid chemical messenger produced by bacteria, could cause dispersion in already formed biofilms of multiple types of bacteria and known as a biofilm dispersal agent. This fatty acid also has growth inhibitory or bactericidal effects, which make it as adjunctive therapy for infection prevention. Moreover, C2DA could improve the efficacy of antibiotics, which are not effective enough against biofilm-associated bacteria, in treating biofilm-associated infections.^11-13^ This molecule could induce the production of EPS destroying enzymes by the microorganisms and also plays an important role in exogenous induction of transition of biofilm bacteria to a planktonic state and disrupts pre-established biofilms.^14,15^ The combination of C2DA with traditional antibiotics could provide a promising mechanism for enhancing the activity of these treatments through the disruption of existing biofilms.^16,17^

Vancomycin is a glycopeptide antibiotic acting at the bacterial cell wall. Although this antibiotic is a treatment agent for staphylococcal infections, it usually fails to treat prosthetic device-related infections caused by *S. epidermidis.*^18^

C2DA in combination with other antibiotics exhibits additive and synergistic effects against bacterial biofilm.^12^ Hence therapeutic interventions through combinations of C2DA and vancomycin could be a new strategy to inhibit biofilm formation or eradicate the already formed biofilm.

Using appropriate drug delivery systems may enhance the delivery of drugs to the site of action and therefore, the antimicrobial efficacy will be improved.^19-21^ Liposomes as drug delivery systems have some advantages. They can deliver oil- or water-soluble bactericidal compounds to a wide range of bacterial biofilms and concentrate antimicrobial agents at biofilms interfaces.^22^ Employing this type of drug delivery system has been proved to become a promising approach.^23^

This study aimed to assess the *in vitro* antibacterial activities of nanoliposomal formulations loaded with vancomycin or/and C2DA against the biofilm formed by *S. epidermidis*. To the best of our knowledge, the approach presented here is novel in combatting bacterial biofilm.

## Materials and Methods

### Materials

Hydrogenated soy phosphatidylcholine (HSPC) was ordered from lipoid (USA). Stearylamine (SA) and cholesterol (Chol) and 2,3,5-triphenyl tetrazolium chlorides (TTC) were purchased from Sigma (St Louis, MO). Vancomycin was obtained from Dana Pharmaceutical Company (Tehran, Iran). C2DA purchased from Santa Cruz (Texas, USA), chloroform, methanol, crystal violet, and glucose monohydrate were provided by Merck (Darmstadt, Germany). Trypticase soy agar (TSA), trypticase soy broth (TSB), and Mueller Hinton broth (MHB) were purchased from Himedia (Mumbai, India).

### Liposomal preparation and characterization

Liposomes encapsulated with C2DA were prepared by the solvent evaporation method. Lipids and C2DA were dissolved in chloroform: methanol (2:1). This part of the formulation was then deposited as a thin film in a round-bottom flask using a rotary evaporator (Heidolph, Schwabach, Germany). The lipid film was hydrated by the addition of deionized water. The lipid phase was composed of HSPC, Chol, SA in the molar ratio of 1:1:0.1. The concentration of C2DA in liposomes suspension was 1 mg/mL.

The dehydration and rehydration method was used for the preparation of liposomes containing vancomycin. In brief, a thin lipid film consisted of HSPC, Chol, SA with lipid fraction of 1:1:0.1 were prepared by the solvent evaporation method and then, the solution contains vancomycin in sodium chloride (2 mg/mL) was added to the lipid film. After the preparation of liposomes, all formulations were extruded repeatedly through 1000, 800, 600, 400, 200, and 100 nm polycarbonate membranes. Formulations were passed at least 11 times through the polycarbonate membrane to produce uniform-sized nanoliposomes. The mean particle size and surface charge of the prepared formulations were determined by Zetasizer (Malvern, Worcestershire, UK) at 25 ± 1°C after suitable dilution.^19^

Encapsulation efficacy of all liposomes was determined by the validated HPLC method. C2DA concentration was quantified using the HPLC method with a mobile phase of acetonitrile-tetrahydrofuran-deionized water (50.4:21.6:28, v/v/v) adjusted to pH 2.5 with phosphoric acid with a C18 column and a flow rate of 1.5 mL/min.^24^ The C2DA peak was detected at 2.32 minutes at a wavelength of 210 nm. Vancomycin concentration was quantified using HPLC with a mobile phase of phosphate buffer-acetonitrile (55:45, v/v) adjusted to pH 7.2 with sulfuric acid with a C18 column and a flow rate of 1 mL/min. The vancomycin peak was detected at 2.5 minutes at a wavelength of 254 nm.^25^

### Determination of minimum inhibitory and minimum bactericidal concentration

The minimum inhibitory concentration (MIC) of agents against bacteria was determined by the broth micro-dilution method according to the standards protocols.^20^ *S. epidermidis* strain DSMZ3270 (DSMZ Cloning, Braunschweig, Germany) was used as a microbial strain. A subculture of this strain was prepared in the TSA medium and stored at 37°C for 24 hours. Next, a suspension from the over-night subculture of this strain was prepared in normal saline to reach and match the 0.5-point of McFarland standard. The stock suspension of bacteria was approximately 10^8^ CFU/mL. The inoculum was prepared from stock suspension at the concentration of 10^6^CFU/mL.

Vancomycin and C2DA solutions were prepared by serial two-fold dilutions in TSB medium. (Start from 2 mg/mL for both vancomycin and C2DA). For different agents, 180 µL of each concentration was added to each well of a microtiter plate (three wells for each concentration). This was followed by the addition of 20 µL of the inoculum. The inoculated microplates were incubated at 37°C for 24 hours before being read. The MIC was determined by using trimethyl tetrazolium chloride (TTC) to each well and then incubating for 30 min at 37°C.

To determine the minimum bactericidal concentration (MBC), broth dilutions that inhibit the growth of a bacterial organism were re-cultured onto TSA and were incubated for 24 hours at 37°C. The MBC was defined as the lowest concentration of the antibacterial agents that revealed no visible colonies on the TSA plate.

### Anti-biofilm activity tests

The effect of different formulations of antibiotics on inhibiting biofilm formation was studied.^26^ Bacterial suspension with a concentration about 2.5× 10^6^ CFU/mL was prepared in TSB from an overnight culture of *S. epidermidis* (containing 0.25% glucose) and 20 µL of the suspension was added to each well of the microtiter plate. After that, 200 µL of each formulation was added per well at the selected concentrations and was incubated for 24 hours. After the incubation process, biofilms were rinsed three times with 200 µL sodium chloride and the remaining biofilms were stained with crystal violet (0.3% for 5 minutes). To solubilize the bounded crystal violet, 200 µL of ethanol (96%) was added in each well. The optical density at 540 nm was determined using a microplate reader (Awareness, Palm City, FL). Tests were done in triplicate and the negative control (untreated) group was included in all cases using TSB medium without formulations and antibiotics to ensure the sterility of the medium during the test. The positive control was microbial suspension added to 3 wells to evaluate biofilm formation and retention during the test.

The quantitative measurement of the OD ratio (ODr) was calculated by dividing the optical density of each antimicrobial agent to the optical density of positive control (native biofilm). This measurement was related to the ability of formulation in inhibiting biofilm formation.

The ability of different formulations on removing bacterial biofilm was also investigated in the present study. Bacterial suspension with a concentration of about 10^8^ CFU/mL was prepared in TSB (containing 0.25% glucose) from an overnight culture of *S. epidermidis*. The bacterial cultures were diluted 1:40 in the same diluent and then the wells were filled with 200 µL of diluted culture and incubated overnight at 37°C. The growth medium was discarded and fresh medium was added every 8 hours. After incubation, bacterial biofilm was attached to the bottom of a 96-well polystyrene microtiter plate. Biofilms were washed with distilled water to discard unbound bacteria. Then, biofilms were treated with different formulations after different times of exposure (24, 48, and 72 hours). After the incubation process, biofilm mass was determined by crystal violet staining assay as mentioned earlier. Each experiment was performed at least three replicates.

To evaluate the efficacy of liposomal formulations, bacterial biofilm was prepared as mentioned above. The initial concentration of liposomal antibiotics was adjusted at the same level as free form. Then, 7-fold serial dilutions of stock concentration were prepared in TSB (containing 0.25% glucose) and each dilution series was tested as described earlier.

Blank liposomes were added to the wells as a positive control to consider the possible effect of the lipids on the biofilm. Negative control was also a sterile medium.

### Statistical analysis

SPSS was used for analyzing differences between ODr. Differences between means were statistically significant if the *P* value < 0.05.

## Results

### Liposomal characterization

The encapsulation efficacy of each formulation is shown in Table 1. According to the results, the encapsulation efficacy for liposomal C2DA was more than 60%. However, the encapsulation rate of vancomycin was approximately 40%.

**Table 1:** Encapsulation efficacy of different liposomal formulations (Mean ± SD, n=3).

**Liposomal formulations**	**Encapsulation** **efficacy (%)**	**Drug-loaded** **concentration** **(mg/mL)**
Vancomycin liposomes	33.9 ± 3.5	0.67
C2DA liposomes	67.3 ± 4.3	0.67
Combination liposomes (Encapsulation efficacy of vancomycin)	41.7 ± 3.1	0.83
Combination liposomes (Encapsulation efficacy of C2DA)	64.5 ± 5.1	0.64

The average size, polydispersity index (PDI), and zeta potential of different liposomal formulations are summarized in Table 2. These data show that the mean sizes in all formula were less than 250 nm and also zeta potential values of them were positive.

**Table 2:** Z-average, polydispersity index (PDI), and zeta potential for each liposomal formulation (mean ± SD, n=3)

**Liposomal formulations**	**Z average (nm)**	**PDI**	**Zeta potential (mv)**
Empty liposomes	164.6 ± 3.5	0.339 ± 0.01	45.2 ± 2.5
Vancomycin liposomes	167.8 ± 1.5	0.197 ± 0.01	37.2 ± 1.1
C2DA liposomes	215.5 ± 8.8	0.114 ± 0.01	40.3 ± 1.6
Combination liposomes	220.2 ± 0.2	0.112 ± 0.1	45.5 ± 0.1

### Determination of MIC and MBC

The MIC and MBC values of vancomycin in free form were 2 µg/mL and 4 µg/mL, respectively. For C2DA in free form, MIC and MBC values were 2000 µg/mL and more than 2000 µg/mL, respectively.

### Biofilm formation studies

**Figure 1:**
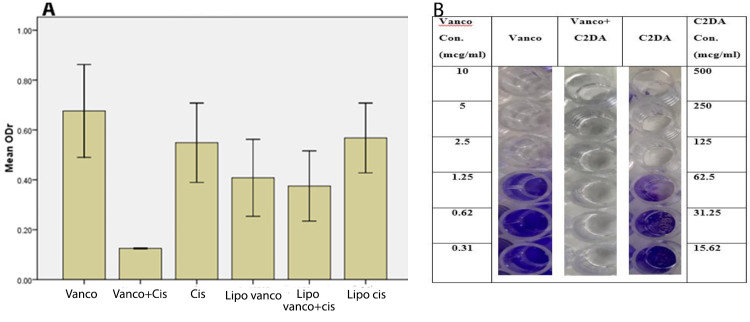
(A) Optical density ratios (ODr) of different wells treated with different formulations are used to inhibit *Staphylococcus epidermidis* biofilm formation. (Mean ± SD, n=3). vancomycin (Vanco). C2DA (cis). combination (vanco+cis). Lipo (liposome). (B) Photographic representation of biofilm formation by *S. epidermidis* in wells of microtiter plates in varying concentrations of C2DA alone and combined (or in combination) with vancomycin. Con (concentration), Vanco (vancomycin)

As shown in Figure 1A, vancomycin and C2DA could not inhibit biofilm formation efficiently while their combination could inhibit biofilm formation completely. Additionally, encapsulation of the antibacterial agents could not improve the ability of them in preventing biofilm formation. These data were also verified by visual inspection of biofilm staining. While the wells of the control group showed complete coverage of all wells, the reduction in staining and coverage of the wells were observed as decreasing the concentration of formulations. By combination, the biofilm formation ability was reduced remarkably (Figure 1B). As seen, C2DA and vancomycin at concentrations of more than 62.5 and 1.25 µg/mL inhibited biofilm formation in microtiter plates, respectively. Upon combination, the sub-inhibitory concentrations were effective in inhibiting biofilm formation.

### Biofilm eradication studies

**Figure 2:**
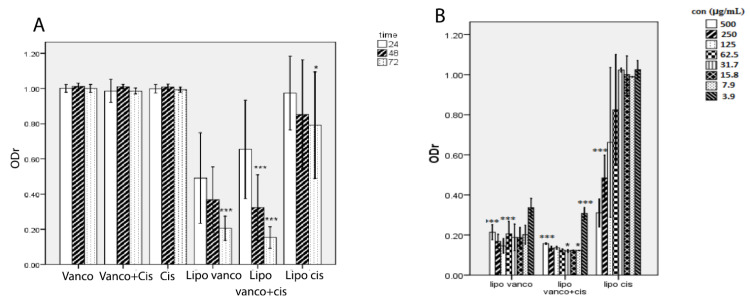
(A) Optical density ratios (ODr) of the C2DA, vancomycin, and combination of them in liposomal and free forms used to eradicate *Staphylococcus epidermidis* biofilm at a different time (Mean ± SD, n=3 or 4), ***P  <  0.001 with respect to 24 h and their free form. Liposomal vancomycin (Lipo. Vanco), liposomal C2DA (lipo.cis), and liposomal combination (Lipo. Vano+cis). (B) Optical density ratios (ODr) of the various liposomal formulations are used to treat *S. epidermidis* biofilm at different concentrations (mean ± SD, n=3).

**Figure 3:**
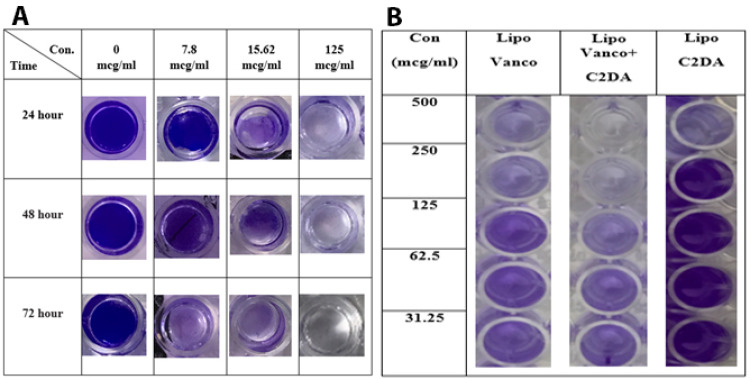
(A) Representative photographs of crystal violet stained wells at various concentrations of the liposomal combination after 24, 48, and 72 hours. (B) Photographic and representation of biofilm after 24 hours in wells of microtiter plates in different concentrations of liposomal formulations of C2DA, vancomycin, and their combination. (The concentration of both C2DA and vancomycin in combination form is the same as their single form). Liposomal Vanco (lipo. Vanco), liposomal C2DA (lipo. C2DA), Concentration (Con).

The ability of antibacterial agents in free and encapsulated forms on biofilm eradication was also studied. As seen in Figure 2, vancomycin alone or in combination with C2DA was ineffective in eradicating the biofilm at 250-fold of MIC values but their liposomal forms could eradicate the biofilm. The liposomal combination was the most effective formulation for biofilm eradicate. Incubation time and concentration of antibacterial agents play a significant role in the efficacy of formulations. During the incubation time, the ability of formulations on removing biofilm was enhanced significantly (Figure 2A). Whereas the reduction in antibacterial agent concentrations led to reducing the efficacy of formulations in biofilm removal (Figure 2B). The photographic representations of these influencing factors are shown in Figure 3.

## Discussion

Microbial biofilm is among the important causes of implant-associated infections which can lead to major medical and economic sequelae. Hence, finding solutions for preventing and treating such infections is becoming more crucial.^7,25^ In the present study, the effects of an antibiotic, vancomycin, and a dispersing agent, C2DA, alone and in combination with each other in both free and encapsulated forms on biofilm formation and eradication of *S. epidermidis* were investigated.

Vancomycin is a glycopeptide antibiotic that inhibits bacterial cell wall synthesis. Previously, it has been demonstrated that vancomycin does not show pronounced effects on prosthetic device-related infections caused by *S. epidermidis,* and for eliminating the biofilm more than 16-fold of MIC values is needed.^4^ Therefore, in the present study, the highest concentration of vancomycin was adjusted at 250-fold of MIC values. The results indicate that vancomycin can prevent bacterial biofilm formation, but, ineffective for the eradication of formed biofilm even at the highest concentration (Figures 1 and 2). These results were in line with previously published data. It was shown that vancomycin is effective only on 6-hour biofilm of *S. epidermidis* and does not affect older biofilms.^27^ Vancomycin is effective only against growing cells and does not show activity against the cells within the biofilm that are not growing or growing slowly.^28^ Farber *et al*. found that exopolysaccharide (EPS) in *S. epidermidis* strains increases the MIC and MBC values of vancomycin, and antagonizes the antimicrobial activity in a concentration-dependent manner. EPS physically complexes with vancomycin and may coat the cell wall and either serve as a barrier to vancomycin penetration or interfere with its action on the cell wall itself.^29^ In another study, the *in vivo* activity of vancomycin was assessed against bacterial biofilm. It was found that, despite the high concentration of used vancomycin, the antibiotic cannot eliminate the biofilm of *S. epidermidis* on the implant surface. This could be due to the high binding of vancomycin to specific components within the biofilm.^30^ Other possible reasons for the low activity of vancomycin against biofilm are the antibiotic molecular interference with the biofilm environment and also the alteration of bacterial metabolism and gene expression due to anaerobic conditions and lack of access to food in the biofilm.^31^ Additionally, antibiotics that inhibit cell wall synthesis are less effective against bacterial biofilm.^32^

In this study, C2DA as a biofilm dispersing agent was used. It was shown that, on one hand, C2DA was able to inhibit biofilm formation and on the other hand, by combining with vancomycin, the synergistic effects were observed and lower concentrations of both vancomycin and C2DA were needed to prevent biofilm formation (Figure 1). Rahmani-Badi et al investigated the effect of exposure to nanomolar concentrations of C2DA on pre-established single- and dual-species biofilms formed by *Escherichia coli* and *Klebsiella pneumoniae* in Petri dish cultures. Treatments with C2DA resulted in a significant increase in the populations of planktonic cells released into the bulk liquid compared with untreated control samples. They also tested the effectiveness of combined C2DA treatments on the removal of pre-established biofilms. They observed that the combination had a significant effect on removing pre-established biofilms.^33^ In a similar study, the effect of various concentrations of C2DA on the biofilm dispersion of *S. aureus*, *Bacillus cereus,* and *Salmonella enterica* was investigated. The most increase in the number of planktonic cells happened with a concentration of 310 nM of C2DA. Moreover, a combination of vancomycin, ciprofloxacin, and ampicillin with C2DA caused a much more reduction in the biofilm mass compared to antibiotics alone. It was found that the combination of ciprofloxacin with C2DA has the highest effect on Gram-negative organisms while vancomycin combination with C2DA eliminated Gram-positive biofilms more effectively.^34^ Marques et al demonstrated that adding C2DA to antibiotics resulted in a significantly greater decrease in the number of resistant cells regardless of the type of bacteria species and growth conditions, compared to antimicrobial treatment alone. They also showed that the combination of antibiotics with C2DA could significantly reduce the number of live cells.^35^ Jenning et al studied the effects of various concentrations of C2DA on inhibiting the formation of methicillin-resistant *Staphylococcus aureus* (MRSA) biofilms. They also investigated the effects of each concentration of C2DA in combination with antibiotics on inhibiting biofilm formation. They demonstrated that the antibiotics at a concentration of 2 μg/mL had an inhibitory effect on MRSA. At a lower concentration (1 μg/mL) when combined with C2DA, there was a synergistic effect in inhibiting growth and inhibiting the formation of biofilm.^11^

Various anti-biofilm mechanisms have been demonstrated for these types of molecules. Masters et alconcluded that the mechanism of action may be used as a response predictor for interaction between C2DA and antimicrobials. ^12^ The structure of C2DA may contribute to its mechanism for incorporating into the bacterial cell membrane and increasing membrane permeability. C2DA is a short-chain fatty acid with a cis bond, which has a bent structure. This structure, along with the amphipathic properties of the molecule, may allow interaction with the phospholipid membrane of bacterial cells. It has been proposed that this interaction could permeabilize the cell membrane.^36^ Therefore synergistic effects could be observed when combined with the antibiotics with intracellular targets such as amikacin, ciprofloxacin, linezolid, and tetracycline. On the other hand, additive effects could be observed when antimicrobial agents act at the same site as bacteria.^12^

The eradication of formed biofilm was another aim of the present study. The effect of C2DA and vancomycin, alone and in combination with each other, on the eradication of formed biofilm produced by *S. epidermidis,*was studied. The data showed that neither vancomycin nor C2DA could cause dispersion in pre-established biofilm. Their combination also fails to eradicate the *S. epidermidis* biofilm (Figure 2). These findings were in contrast with previous studies that analyzed the effect of C2DA on the biofilm formed by *E. coli* and *K. pneumonia*^33^ and *P. aeruginosa, E. coli, Proteus mirabilis, Streptococcus pyogenes, Bacillus subtilis, S. aureus,*and* Candida albicans.*^16^ These contradictory results may be due to the different bacteria that are used for biofilm formation. It has also been noted that antibiotic penetration into biofilms depends on the type of biofilm and antibiotic.^37^ Therefore, we may conclude that the penetration of the tested compounds into the *S. epidermidis* biofilm was low.

Another explanation for this contradictory result could be the age of biofilm. Monzon et al studied the correlation between the age of biofilm and efficacy of a different antibiotic, even individual or in combinations, on the biofilms of *S. epidermidis*. They found out that the effect of antibiotics combinations on biofilm eradication increased as the age of biofilm decreased. The inefficacy of antibiotics like vancomycin in older biofilm could be because of the slow growth of biofilm bacteria, which may make the microorganism less susceptible to antibiotics. It could also be due to low antibiotic penetration through biofilm layers. Because of the high molecular weight of vancomycin and its high solubility in water, it would accumulate in biofilm but could not reach or affect the deep layers of biofilm.^37^

As another approach, the antibacterial agents could be encapsulated with drug delivery systems for better interaction.^38^ In this study, the liposomal formulation containing vancomycin, C2DA, and a combination of them was prepared and used. The results of physicochemical properties evaluation (Tables 1 and 2) indicated that the encapsulation efficiency values were suitable for both antibacterial agents and the particle size diameters were also in line in a good range for delivery to bacteria. The effect of liposomal formulations on biofilm formation was evaluated and the results indicated that the effectiveness of formulation, except liposomal vancomycin, was not improved with respect to the free form. The indifference activity of encapsulated with the free form of antibacterial agents might be due to the lower interaction of bacteria and molecules in encapsulated forms. In the liposomal form, due to the slow release of the components into the environment, not all components may release into the environment during the incubation period. Moreover, only at high concentrations, the formation of biofilm was inhibited. The indifferences antimicrobial activities of encapsulated agents with respect to the free form at initial incubation time were previously described.^21^

In the field of biofilm eradication studies, based on the findings, although free forms could not eradicate biofilms at all, the liposomal forms result in a significant decrease in pre-established biofilms at the same concentrations as the free forms. The efficacy of liposomes as a drug delivery system is due to their absorption into the cell wall of the bacteria. As a result, the drug’s effectiveness depends on absorbing the liposome into the surface of the bacterium.^38^ The choice of appropriate lipids with a proper concentration in the preparation of liposomes could have an important effect on liposomal absorption to the cell surface. It has been shown that the use of cationic lipids in liposome preparation can be effective against *S. epidermidis* biofilm.^22^ Jones et al showed that the most effective systems for bactericide delivery to *S*. *epidermidis*biofilm were DPPC-cholesterol-SA liposomes. The adsorption of the liposome carrier to the bacterium surface could be facilitated by the introduction of ionic interactions in the case of cationic liposomes incorporating SA.^39^

Based on the previous results, the interaction between cationic liposomes and biofilm was stronger than others. Anionic liposome had a lower absorption due to repulsion with bacterial cells.^40^ The stronger attachment causes the cationic liposomes to be in direct contact with the biofilm surface. Therefore the released contents would have a greater chance of diffusing into the biofilm than the free drug in solution. In our study, SA was used to prepare cationic liposomes. Liposomes can also protect the encapsulated drug from binding to the EPS components or inactivation by enzymes and thus can increase the antimicrobial effect of the drug compared with the free form.^41^

The best results were observed for liposomal combination, and the effectiveness of the formulations was increased by time (Figure 2). These observations could be due to the effect of formulation components. A study has shown that the higher the level of cholesterol in the liposomal formulation led to faster release of the hydrophilic drug and slower release of hydrophobic ones by creating spatial inhibition.^42^ As a result, the presence of cholesterol in lipid formulation could be effective in observed findings. Vancomycin is a hydrophilic molecule, and C2DA is a hydrophobic compound. During the first 24 hours, C2DA may not be fully released into the environment due to its slow rate of release, and therefore no synergistic effect will occur on biofilm degradation. Consequently, after 24 hours of incubation, the efficacy of the combined liposomal formulation was not superior to the vancomycin liposome. However, after 48 and 72 hours, C2DA was completely released to the environment, and, as a result, the efficacy of the combination form is greater than vancomycin alone. According to Moghadas-Sharif et al, incubation time plays an important role in the efficacy of the liposomal formulation. As the incubation time increases, the biofilm eradication rate is increased. In their study, vancomycin-containing liposomes had the highest effect on *S. epidermidis* biofilm in 72 hours and the lowest effect in 24 hours.^7^

Sanderson showed the effectiveness of the cationic liposomes encapsulating vancomycin against S.*epidermidis* biofilms. The results indicated that by increasing the duration of incubation, the effect of vancomycin liposomal in inhibition of bacterial growth is increased. By increasing incubation time, the encapsulated contents are more likely to leak out.^43^

In the present study, by lowering the concentration of formulations, their efficacy on biofilm inhibition and eradication has been decreased especially in the liposomal form (Figures 2 and 3). In one study, to evaluate the importance of concentration in antibiotic efficacy, serial two-fold dilutions of liposomal formulations have been done and their efficacy against the biofilm of *S. epidermidis* was investigated.^7^ This was also proposed by Monzón et al, which showed that antibiotics concentration and exposure time affected the efficacy of antibiotic treatment in biofilms. Increasing the exposure time and antibiotic concentration either individually or together increases the efficacy of treatment on *S. epidermidis* biofilm.^37^

## Conclusion

Incorporating C2DA and vancomycin that act on the cell wall, into a drug delivery system such as liposome could eradicate biofilm and decrease the risk of implant-associated musculoskeletal infection. Further studies related to the action of the anti-biofilm agent, C2DA, with various antibiotics are necessary to develop a potential clinical therapy, which is effective in completely inhibiting biofilm growth or eradicating biofilm at implant surface.

## Acknowledgments

The results described in this article were part of a Pharm. D. student thesis. This work was supported financially by a research grant from the Vice Chancellor for Research of Mashhad University of Medical Sciences, Mashhad, Iran.

## Conflict of Interest

The authors declare that there is no conflict of interest in this study.

## Ethical Issues

Not applicable.
